# Obtaining a faculty position at a primarily undergraduate institution (PUI)

**DOI:** 10.1186/s12919-021-00207-6

**Published:** 2021-06-22

**Authors:** Christina King-Smith, Caroline (Lina) Lund Dahlberg, Blake Riggs

**Affiliations:** 1 Saint Joseph’s University, Philadelphia, PA USA; 2 Western Washington University, Bellingham, WA USA; 3 San Francisco State University, San Francisco, CA USA

**Keywords:** PUI, Life science careers, Primarily undergraduate institution

## Abstract

Scientists who hope to obtain a faculty position at a primarily undergraduate institution (PUI) need a distinct skill set and outlook on their future teaching and research career. To obtain a position at a PUI, candidates should 1) design a strategy for obtaining a faculty position that suits each individual’s career goals and aspirations, 2) prepare for the application process, on-campus interview, and contract negotiations, and 3) plan a strategy for the probationary period leading up to tenure and promotion. Given the different types of PUIs, candidates need to consider whether they seek a position that consists of all or mostly all teaching, or both teaching and research. Candidates should educate themselves on the expectations at PUI’s, including current thought, practice, and aspirations for science pedagogy, and gain teaching experience prior to seeking a suitable position. If the candidate’s goal is a position with both teaching and research, it is important to discuss with the current research mentor what projects the candidate can take with them to their new position. The candidate should also consider what types of projects will be successful with undergraduate student researchers in a PUI research environment. Importantly, candidates should clearly demonstrate a commitment to diversity and inclusion in their teaching, research, and outreach, and application materials should demonstrate this. On interviews, candidates should be knowledgeable about the mission, values, and resources of the institution and how the candidate will contribute to that mission. Once hired, new faculty should discuss a formal or informal mentoring plan during the probationary period that includes peer evaluations on a regular basis, and maintain communication with the department chair or designated mentor regarding teaching, research, and service activities.

## Background

### Why work at a primarily undergraduate institution?

Scientists who have positions at primarily undergraduate institutions (PUIs) have at least one commonality: they are passionate about teaching and learning with undergraduate students. Whether interested in a teaching-only position, or a blend of teaching and research with undergraduate students as collaborators, teaching and mentorship are of primary importance at a PUI. The rewards of research with undergraduate students include not only the satisfaction that accompanies research and scholarship, but also the opportunity to engage undergraduate students in hands-on learning as research collaborators. Prospective PUI faculty must recognize that at a PUI the pace of research will be much slower than at a research institution, and that the research resources available at PUIs may not be what is available at a research institution. A faculty position at a PUI is not a “fall back” job for scientists who aspire to careers at research universities. Rather, it requires dedication to student learning and a skill set that includes excellent teaching, mentorship, research prowess, and the ability to manage the often conflicting time demands of teaching, research, and service. Nevertheless, with preparation, realistic expectations, resilience, and good mentoring, faculty can derive much professional satisfaction at a PUI.

This paper summarizes three main stages of obtaining a position at a PUI: identifying, preparing, and applying for a position; the interview process and negotiation, and achieving success during the probationary period leading to tenure and promotion.

### Design a strategy for obtaining a faculty position that suits each individual’s career goals and aspirations

#### Know what kind of position you seek

Not all PUIs have the same focus, so it is important for candidates to reflect on what their ideal position entails. Faculty at some PUIs, including some four-year colleges, community colleges and junior colleges, will spend nearly all their time teaching, with no expectations for research, and minimal or no support for faculty desiring to conduct research. Other institutions view faculty research as an important component of undergraduate education, and will support and expect faculty research and scholarship by providing dedicated research space, start-up funds, faculty development resources and more, with the expectation that faculty will include and mentor undergraduate students in research.

#### Know the expectations

Candidates seeking a PUI position must scrutinize job advertisements carefully, and tailor their application to fit the expectations of each particular position. Faculty at PUIs will carry a much higher teaching load than a faculty member at a research institution, and requirements for tenure and promotion will weigh heavily on the individual’s teaching ability and success. While teaching loads will vary depending on the institution, generally positions that require research will have a lighter teaching load than institutions having no research requirement. For example, at a four-year institution where there is an expectation of research, faculty may be responsible for the equivalent of three courses each semester, meeting three times a week (nine “contact hours”), in addition to drop-in or by-appointment office hours each week. Mentoring students in research may or may not count towards the teaching load. At a teaching-only institution, faculty may teach four courses a semester, or four (or more) courses that are combinations of lectures and laboratories. At most institutions, regardless of whether there is a research expectation, faculty are also expected to participate in service, such as serving on department, college, and university committees, acting as a faculty advisor for a student group, or performing professional service or science outreach.

Because teaching is central to the mission of PUIs, faculty candidates should familiarize themselves with current evidence on undergraduate STEM education. “Vision and Change in Undergraduate Education: A Call to Action” and a follow-up report, “Vision and Change: Unpacking a Movement and Sharing Lessons Learned [1, 2] encapsulate current thought on transforming undergraduate education, through practices such as evidence-based, student-centered, active learning that integrates core ideas across biology courses, emphasizes depth over breadth, and is inclusive of all students. Many scientific professional societies have subcommittees focused on science education, and have education sessions at their annual meetings where prospective PUI faculty can learn about current science pedagogy ideas and language, such as “experiential learning”, “flipped classroom”, clicker questions, just-in-time teaching, etc. Science pedagogy journals such as *CBE- Life Sciences Education,* published by the ASCB, are also useful resources. In addition to understanding how these active learning techniques can benefit students, applicants should also think critically about how to build inclusive teaching practices into their classrooms and teaching (see, for example, [3, 4].

#### Gain teaching experience

It is imperative that prospective PUI faculty gain college-level teaching experience in some form, to learn about developing course materials and teaching style, connecting with students, grading expectations, and to become familiar with online learning management systems used by most colleges and universities. Occasionally, candidates who have outstanding equivalent experience, such as an intensive teaching workshop, outstanding research expertise, or specific skills, will be successful landing a position at a PUI without prior teaching. However, the majority of PUIs will expect some college-level teaching experience. For graduate students, serving as a Teaching Assistant is an excellent entry to teaching. For postdoctoral fellows, a semester or more as a part-time, adjunct/affiliate faculty, either for science majors or non-science majors, is valuable preparation. Another option is a temporary, full-time position as non-tenure track, Visiting Faculty. These positions are generally for one academic year and may be renewable. In some cases, Visiting Faculty positions may be converted to tenure-track positions, but this is normally accompanied by a full search process for the new position.

For adjunct or Visiting Faculty interested in full-time teaching at a PUI in the future, it can be helpful to discuss career goals with the department chair. Most departments are required to do peer evaluations of adjunct/affiliate faculty, and many department chairs and faculty are happy to help aspiring PUI faculty by conducting evaluations and offering constructive feedback. Excellent bench and field research skills do not necessarily translate to excellent communication skills for undergraduate teaching, and peer evaluation by experienced PUI faculty can help identify areas for improvement. Even if not required, request a peer evaluation, which should include both review of class materials (quizzes, exams, laboratory handouts, syllabi, etc.) and a classroom visit. If the department lacks a standard evaluation rubric, the Classroom Observation Protocol for Undergraduate STEM (COPUS; ref. [5]) can provide valuable feedback on active learning and teaching.

With the advent of the Covid-19 pandemic in spring of 2020, faculty at colleges and universities had mere days to plan and execute online delivery of their courses, mid-term. While the expectation is that post-pandemic, university classrooms and labs will be back to “normal”, the “normal” will likely change, thus having experience in teaching science online, or teaching in a hybrid online/“on ground” format, will similarly strengthen an application. While faculty positions may only advertise or expect on-ground teaching, with a decrease in the number of college-ready students in the coming years [6], many colleges and universities are competing for the same students. Departments that are able to offer both online and “on-ground” classes can potentially attract more students, and more types of students, such as adult learners. On-line learning can also increase inclusion, and can enable students who may have difficulty attending courses on-ground to complete bachelor’s degrees (see, for example, [7]). Thus, faculty able to teach effectively in either modality may be more attractive to employers, and in coming years, teaching at least some classes online or in a hybrid format may be an expectation.

Graduate students interested in a PUI position combining teaching and research may wish to consider postdoctoral fellowships that have specific opportunities for teaching. For example, the NIH-supported Institutional Research and Academic Career Development Awards (IRADCA), allow postdoctoral fellows at research-intensive institutions to partner with PUIs having a commitment to training students who are underrepresented in biomedical science.

#### Research with undergraduate students

Running a research laboratory and mentoring undergraduate or masters level research students are generally not taught at the graduate or postdoctoral level, yet these are important skills for PUI faculty candidates. One excellent learning opportunity, where feasible, is for graduate students or postdoctoral fellows to mentor undergraduate students in their research mentor’s laboratory. This can enrich a candidate’s portfolio, especially if the mentoring leads to conference presentations or publications with undergraduate student co-authors.

When planning a job search, prospective PUI faculty should discuss with their Principal Investigator what research they will be able to take with them to their new position, and, if appropriate, what research collaborations might be possible. Collaborations can be a positive indicator for faculty tenure and promotion, however faculty will likely be required to show evidence that their part of the collaboration can stand on its own. Prospective PUI faculty should reflect on how, specifically, undergraduate students will participate in their research. While projects that are technically difficult are possible with undergraduates, keep in mind that undergraduate students are *very* busy, with four to five classes per semester (typically including two courses with accompanying labs) and extracurricular activities. Large blocks of time for research may not be possible for undergraduates during the academic year. Several institutions have summer research programs, and prospective faculty should inquire about this, since having students full-time in the summer months can result in much research progress.

Another option for incorporating research into undergraduate education is through Course-Based Undergraduate Research Experiences (CUREs). These are courses built around an open-ended, research project [8–10]. Importantly, course-based research can augment work done in a faculty lab, and can lead to student-authored publications.

While PUIs will likely have some form of internal research support, gaining extramural grant support will further enhance your ability to do research. Become familiar with extramural grant opportunities, for instance, NSF CAREER grants for new investigators, and especially those grants targeted to PUIs, such as the NSF Research in Undergraduate Institutions (RUI) awards. NSF also offers Research Experiences for Undergraduates (REU) Supplements, which provide additional funds for new or renewal NSF awards. NIH offers the R15 Academic Research Enhancement Awards (AREA) program. This has been reorganized to include two branches, one, the AREA for Undergraduate-Focused Institutions, is appropriate for funding research at PUIs.

### Prepare for the process of applying for a faculty position, on-campus interview, and negotiating an employment contract

#### Completing the application

Advertisements for positions at PUIs can be found at numerous online sites (the *Chronicle of Higher Education*, HigerEdJobs), journals such as *Science* and *Nature,* American Society for Cell Biology (ASCB) and other professional society websites. When applying for a position, it is critical to read the job ad carefully. The cover letter should directly address the needs of the department. Many PUIs seeking faculty have concrete needs in terms of teaching, but prefer to cast a wide net in terms of research to find a candidate who is not only an excellent teacher but is doing exciting research with promise for undergraduate involvement. Read the department website to determine if core facilities needed to support research (animal, microscopy, tissue culture facilities, etc.) are available. Regarding spousal accommodations, while they are often offered at research institutions, they are rare at small PUIs. However, a PUI in a major metropolitan area with multiple institutions may offer opportunities that accommodate a partner’s career.

If asked for statements on teaching philosophy and research, include them. The teaching statement need not be many pages, but should convey enthusiasm and interest in teaching undergraduate students. Some mention of teaching strategies could be included here. Some institutions may request evidence of teaching excellence, including peer evaluations, sample syllabi, or select or summarized student evaluations. If the position requires research, the teaching statement should also outline how students will be involved in your research program. A separate statement describing your research area may also be requested, and should be understandable to a generalist in the discipline. Many PUI departments contain faculty with a wide array of specialties; so it is best to avoid jargon only understandable by someone in your sub-field.

#### Diversity and inclusion

PUIs are increasingly, and appropriately, devoting much effort to ensuring that teaching is inclusive and equitable. This involves understanding instructional practices ranging from active learning approaches to inclusive language [3, 11]. Some institutions will ask for an additional statement describing your commitment to diversity, equity and inclusion (DEI). If a DEI statement is not specifically requested, candidates should address this in their teaching statement. The statement should include the candidate’s experience and training in this area, including teaching, mentoring or any certificate programs. Seek ways to connect ideas in a DEI statement to the mission of the institution. If the institution has a DEI office or committee, discuss potential ways of interacting or collaborating. Many institutions provide professional development surrounding DEI classroom practices, and having knowledge about what is offered and how these practices are connected to the department will strengthen the application.

#### The interview

Many faculty search committees make a short list of candidates for a phone or video conference interview prior to inviting finalists for an on-campus interview. Candidates should prepare for a phone interview by re-reading the job ad and reviewing the institution and department website to become familiar with the mission and vision of the institution, and have a general idea of the curricular requirements. Review the department faculty pages and research interests, especially noting potential research collaborators. Be prepared to discuss what existing and new courses you could teach, and expect questions such as the strengths you could bring to help the department achieve their goals; challenges you would face, and why you are attracted to that specific institution. Compose a list of questions; ask how faculty research is supported, and, if applicable, be prepared to discuss what external funding you will seek.

For the on-campus interview, review the institution’s website more intently to learn more about the faculty in the department, their teaching responsibilities, research areas, and publications. Become familiar with the curriculum, including required introductory and upper division or capstone courses. Candidates will likely be asked to present a research seminar or teaching demonstration at the interview. If the former, be sure to target the seminar to the audience, likely undergraduate students and faculty who are not specialists in your field. The seminar or teaching demonstration should showcase your ability to communicate and engage your audience, and (if applicable) should include some specific examples of research projects in which undergraduate students will participate.

For a position requiring research, candidates may be asked for a list of equipment needs. The more specific the list, the better. The candidate should ask what startup funds are available for new faculty, what common equipment or instrumentation is available, and what instrumentation is needed but might already be available in another faculty lab or teaching lab. Other important questions to ask at the interview are the expectations for teaching and research, including the number of courses taught per semester. Does the institution have a teaching and learning center, or other support for improving teaching? Are there faculty professional development resources to support travel to research conferences, research funds available after the start-up period? Is there an extramural research office that will support grant writing? Release time from teaching if faculty secure extramural funding? What is the sabbatical policy? Is there a pre-tenure sabbatical? What is the institution’s policy on family medical leave?

Faculty should also ask the department how pre-tenure faculty are mentored. Some departments have a formalized mentoring system; in other departments it may be more loosely structured. Other questions to ask regarding the probationary period: how does peer-evaluation work? What is the “department culture” regarding peer evaluation? How do student course evaluations, research with students, service, etc., contribute to faculty evaluation for tenure and promotion?

#### The offer: negotiation

When presented with a job offer the candidate should be very clear on what the teaching and advising responsibilities are, salary and benefits, and the laboratory start-up package. Institutions will vary on how much flexibility a dean has to negotiate salary and start-up funds. More flexibility in negotiation may be possible regarding start-up. For example, the candidate may be able to negotiate purchase of an instrument that can be used by several other faculty, and separate that purchase price from the start-up budget. Consulting colleagues who are new hires at PUIs on their packages can be informative, however institutions are not all alike. One institution may offer a higher salary, but another might have richer benefits, such as a more generous retirement and health plan, tuition benefits for family members, or valuable but less tangible benefits, such as a preferred geographical location, or a mission that especially resonates with the faculty candidate (see the accompanying article, Dahlberg, et al.,  for more information on start-up negotiation).

#### Plan a strategy for the probationary period leading up to tenure

Institutions invest much time, effort, and expense in recruiting and hiring the best candidates, therefore it is in the institution’s best interest that new faculty succeed. However, depending on the department and institution, and how formal the probationary period evaluation is, the probationary faculty member may need to be proactive about seeking peer evaluations, advice on teaching and mentoring research students, and how to balance teaching, service, and research. Once a position is secured, faculty should discuss a mentoring plan with the department chair (for an overview of mentoring practices, see [12]). Review the institution and departmental guidelines on promotion and tenure. The expectation is that new faculty will improve their teaching in the first few years, and pre-tenure faculty should take advantage of faculty development resources available at the institution. In terms of research, many institutions have department-specific guidelines that will help tenure and promotion committees interpret a candidate’s success and progress in their field. Regular communication with a mentor and department chair will insure that the candidate is making acceptable progress in both teaching and research that lead to tenure and promotion.

Expect to spend **much** time teaching the first year. It is not uncommon to spend three hours or more in preparation for each one-hour lecture, and possibly more for laboratory sections, in addition to writing and grading exams, reading student papers, meeting with students for office hours, advising, etc. Establishing a research lab and starting to formulate research grant ideas should also occur in the first year (see accompanying article, Dahlberg, et al.). Clarify expectations for student researchers, and think carefully about the number of students you can effectively mentor as a first-year faculty member.

Work with the chair to determine what level of service is expected, and explore what service opportunities are available and of interest to you. Not all committees demand the same time commitment, so it is important to discuss service opportunities with senior faculty and the department chair. Generally new faculty serve on department committees early in their career, and once they are more familiar with the institution, may move into college or university-level committees.

## Conclusions

A position at a primarily undergraduate institution can be immensely rewarding for scientists who enjoy interacting with students, mentoring students, and are passionate about teaching. While the pace of research at a PUI is slower compared a research institution, research with undergraduates is not only rewarding to the mentor, but can also produce meritorious and rigorous scholarship while allowing students to actively participate in a research experience. The conflicting demands of teaching, research, and service are challenging to PUI faculty, but with planning, preparation, and effective mentoring, young scientists can thrive and gain much satisfaction in a career at a PUI.

## Data Availability

Not applicable.

## Abbreviations

ASCB: American Society for Cell Biology
COPUS: Classroom observation protocol for undergraduate STEM
IRADCA: Institutional research and academic career development awards
CURE: Course-based undergraduate research experience
DEI: Diversity, equity, and inclusion
NSF: National Science Foundation
NIH: National Institutes of Health
RUI: Research in undergraduate institutions
PUI: Primarily undergraduate institution
REU: Research experiences for undergraduates
STEM: Science, technology, engineering, and mathematics
